# The effect of a dynamic PCL brace on patellofemoral compartment pressures in PCL-and PCL/PLC-deficient knees

**DOI:** 10.1186/s40634-017-0085-5

**Published:** 2017-03-31

**Authors:** Tyler Welch, Thomas Keller, Ruben Maldonado, Melodie Metzger, Karen Mohr, Ronald Kvitne

**Affiliations:** Kerlan Jobe Orthopaedic Clinic, Los Angeles, CA USA

**Keywords:** Peak pressure, Posterior cruciate ligament, Posterolateral corner, Patellofemoral joint

## Abstract

**Background:**

The natural history of posterior cruciate ligament (PCL) deficiency includes the development of arthrosis in the patellofemoral joint (PFJ). The purpose of this biomechanical study was to evaluate the hypothesis that dynamic bracing reduces PFJ pressures in PCL- and combined PCL/posterolateral corner (PLC)-deficient knees. Study Design: Controlled Laboratory Study.

**Methods:**

Eight fresh frozen cadaveric knees with intact cruciate and collateral ligaments were included. PFJ pressures and force were measured using a pressure mapping system via a lateral arthrotomy at knee flexion angles of 30°, 60°, 90°, and 120° in intact, PCL-deficient, and PCL/PLC-deficient knees under a combined quadriceps/hamstrings load of 400 N/200 N. Testing was then repeated in PCL- and PCL/PLC-deficient knees after application of a dynamic PCL brace.

**Results:**

Application of a dynamic PCL brace led to a reduction in peak PFJ pressures in PCL-deficient knees. In addition, the brace led to a significant reduction in peak pressures in PCL/PLC-deficient knees at 60°, 90°, and 120° of flexion. Application of the dynamic brace also led to a reduction in total PFJ force across all flexion angles for both PCL- and PCL/PLC-deficient knees.

**Conclusion:**

Dynamic bracing reduces PFJ pressures in PCL- and combined PCL/PLC-deficient knees, particularly at high degrees of knee flexion.

## Background

The natural history of posterior cruciate ligament (PCL)-deficiency includes significant knee pain and arthrosis in the medial and patellofemoral compartments (PFJ) (Wijdicks et al. 2013; Kennedy et al. 2014; LaPrade et al. 2015a) (Gill et al. 2003b; Kennedy et al. 2013; Patel et al. 2007; Shelbourne et al. 2013; Strobel et al. 2003; Torg et al. 1989). The exact mechanism of articular cartilage degeneration in PCL-deficient knees remains unknown; however, several cadaveric studies have reported that PCL deficiency leads to a significant increase in contact pressure in these two knee compartments (Gill et al. 2003b; Grood et al. 1988; Markolf et al. 1993; Strobel et al. 2003). This increase in compartmental pressure is possibly the result of increased anterior–posterior laxity (MacDonald et al. 1996; Anderson et al. 2012; Fanelli & Edson 1995; Gill et al. 2003b; Goyal et al. 2012; Kumagai et al. 2002; Logan et al. 2004) and rotational instability (Jonsson & Karrholm 1999 Gill et al. 2003a; Kennedy et al. 2013) of the knee. PCL injuries rarely occur in isolation, and concomitant posterolateral corner (PLC) injuries are common, particularly in a trauma setting (Fanelli & Edson 1995). The PLC resists excessive varus and external rotation forces in the knee (Markolf et al. 1993; Torg et al. 1989). The PLC also plays a secondary role in resisting posterior translation of the tibia. Therefore, the PLC and PCL play a symbiotic role in resisting excessive external rotation and posterior translation of the proximal tibia.

While optimal treatment of isolated PCL and multi-ligament knee injuries is unclear, management may include bracing to restore posterior and rotational stability in the knee. Static braces provide a constant anterior force through the entire arc of knee range of motion (Pierce et al. 2013; Jansson et al. 2013a). Several authors have evaluated the effectiveness of static bracing for the treatment of PCL injuries (Ahn et al. 2011; Jung et al. 2008; Spiridonov et al. 2011). While static braces reportedly contribute to satisfactory outcomes, Jacobi et al. demonstrated that appropriate stability is not fully restored following management with a static brace (Jacobi et al. 2010).

Tension within the PCL varies through the knee arc of motion. For instance, forces through the PCL have been shown to increase almost linearly with knee flexion angle (Markolf et al. 2006). Unlike static braces, dynamic PCL braces are designed to provide increased anterior force and improved posterior stability at higher degrees of knee flexion, thus better replicating the natural role of the PCL (Jansson et al. 2013a). In the only study comparing the effect of static versus dynamic bracing on PCL-deficient knees, Laprade et al. demonstrated that dynamic braces due in fact provide more stability than static braces at higher degrees of knee flexion (LaPrade et al. 2015b). By improving knee kinematics, dynamic braces may help normalize medial and PFJ pressures in PCL-deficient knees and potentially reduce the incidence of knee arthrosis. We are not aware of any clinical study that has evaluated peak pressures in the knee or the incidence of arthrosis in PCL- or PCL/PLC-deficient knees treated with a dynamic brace.

The purpose of this biomechanical study is to evaluate peak PFJ pressures in PCL-deficient and PCL/PLC-deficient knees with and without application of a dynamic brace. We hypothesiz that dynamic bracing of PCL- and PCL/PLC-deficient knees will significantly reduce peak pressures in the PFJ, particularly at higher degrees of knee flexion.

## Methods

### Specimen preparation

Ten fresh frozen cadaveric knees (proximal femur through foot) were procured from an institutional-approved tissue bank. Specimens with evidence of injury or instability by physical examination were excluded. All specimens were stored at − 30 °C until testing, at which point they were thawed at room temperature for approximately 24 h.

**Fig. 1:**
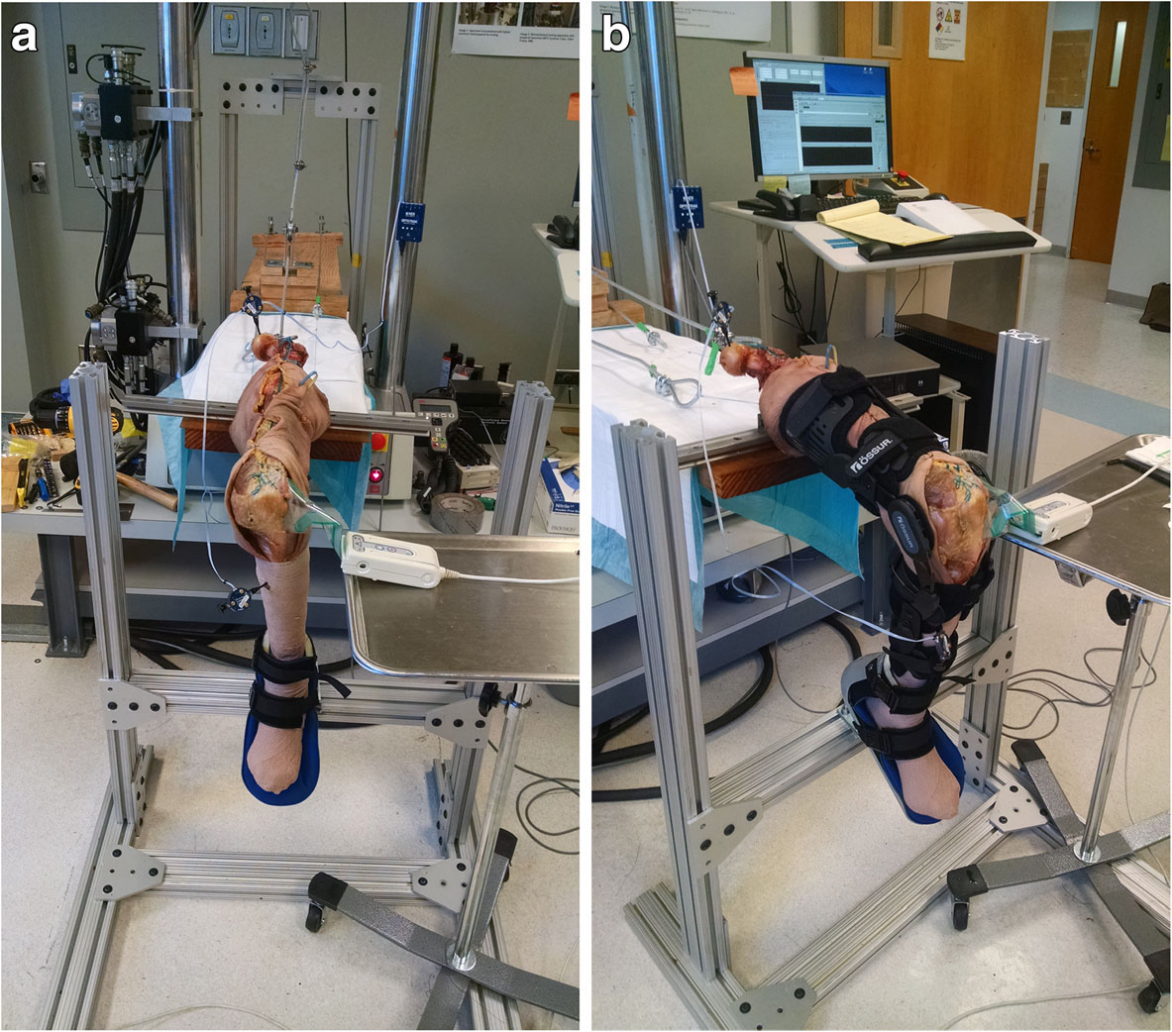
Photographs of design apparatus with cadaveric knee without (**a**) and with (**b**) application of the dynamic brace. Tekscan sensors connected to the handle via a lateral arthrotomy. Weights are attached to suture pulleys to simulate muscle loading through the hamstrings. The quadriceps is attached to the MTS machine via suture to simulate muscle loading

After defrosting, the quadriceps and hamstring tendons were dissected and sutured (#2 Fiberwire, Arthrex, Naples, FL) with locking Krackow stitches just distal to the musculotendinous junction. A custom aluminum stand was designed to hold the knee at 30°, 60°, 90°, and 120° of flexion. The proximal femur of each specimen was also dissected and clamped to the testing frame, while the foot and ankle were placed in a modified ankle foot orthosis (AFO) and secured to the custom-designed aluminum stand using a strap (Fig. 1). The ankle was maintained at 0° of dorsiflexion throughout testing with two tight straps that kept each heel seated in the neutrally-positioned AFO. Sutures from the hamstrings and quadriceps muscles were attached to cables to allow the application of simulated muscle forces. The skin and muscles of the specimen were preserved, and the skin was re-approximated with sutures following dissection to ensure that the brace would fit each specimen appropriately (Fig. 1).

### Contact pressure measurements

PFJ peak contact pressures and total forces were measured with Tekscan pressure mapping sensors (K-Scan 5051, Tekscan Inc., Boston, MA). The 5051 sensor is a 0.1 mm thin, flexible film with printed conductive ink that measures forces with a resolution of 1,936 sensing elements within a 55.9 mm × 55.9 mm sensor matrix area. The sensor is capable of measuring contact pressures up to 8 MPa. Prior to testing, the sensors were reinforced with vinyl laminate and then preconditioned, equilibrated, and calibrated according to the manufacturer’s recommendations.

Sensors were reinforced with vinyl laminate to prevent shear force damage and reduce drift. Once laminated, the sensor was preconditioned using a 2 MPa cyclic load for 30 cycles inside the Tekscan equilibration device, which applied a uniform pressure to the sensing matrix area through an air-filled bladder. In addition, a three-point equilibration process was performed to account for sensing element variation at 50, 100, and 150 raw digital outputs. After equilibration, the sensor was calibrated using a Mechanical Testing System (MTS Bionix 370.02, MTS Corp., Eden Prairie, MN) by applying incremental loads from 0 to 750 N to the sensor. The sensor was compressed between a metal plate and a flat high-density polyethylene block with a 1.5 mm thick silicon rubber sheet below to evenly distribute loads, covering approximately 75% of the sensor matrix area. The raw digital output was then correlated to contact pressures using a power law curve to best fit the non-linear sensor behavior.

### Mechanical testing

Motion tracking cameras (Optotrak Certus, Northern Digital Inc., Waterloo, Ontario, Canada) were used to validate proper angles of the tibia relative to the fixed femur prior to loading. Both tibial and femoral anatomical axes were pre-defined using a digitizing probe. Two infrared diode sensors were placed on both the femur and tibia to track their relative 3D motion.

Once calibrated, the 5051 sensor was placed in the PFJ via a lateral arthrotomy and sutured to the distal quadriceps tendon. A quadriceps load of 400 N was applied via the MTS machine, and a separate load of 200 N was applied to the hamstrings (100 N to biceps femoris and 100 N to semitendinosus/gracilis) using free weights attached to cables. These muscle loads have been used in multiple previous studies evaluating various biomechanical effects of PCL deficiency (Li et al. 2003; Li et al. 2002).

The integrity of the PCL was confirmed by the senior author via posterior drawer test and through visualization during mechanical testing. Two cadavers with PCL insufficiency were excluded. The PCL was cut via a lateral arthrotomy and testing was performed at 30°, 60°, 90° and 120° both with and without a dynamic brace (Ossur Rebound PCL Brace, Ossur, Reykjavik, Iceland) under the simulated muscle loads. Sectioning of the PCL was confirmed visually and via posterior drawer examination. Only specimens with a Grade III Posterior Drawer Test were included. Afterwards, the PLC was cut via the lateral arthrotomy and testing was repeated. Care was taken to preserve the skin and muscle bulk of each specimen so that the brace fit each specimen appropriately.

Each Ossur Rebound brace was custom-fitted to each individual specimen. The Ossur Rebound PCL Brace has three settings; the highest tension setting applies approximately 54.5 Newtons of force to the proximal tibia with the knee in full extension. We used the highest-tension setting for each cadaver.

### Data and statistical analysis

Deep patellofemoral force and area of the applied force was recorded for each knee. Total pressure was calculated as force divided by area. Patellar pressure data were plotted in two dimensions to identify peak pressure areas. A cluster of 16 pixels at the point of maximal peak pressure was determined and averaged to calculate peak pressure values for each testing condition and at each angle (30°, 60°, 90° and 120°).

Multiple repeated measures ANOVA (SAS 9.4, SAS Institute, Inc., Cary, North Carolina) was applied to force, total pressure, and peak pressure for each condition tested (−PCL, −PCL + brace, −PCL/-PLC, −PCL/-PLC + brace), at all four flexion angles tested (30°, 60°, 90°, and 120°), and for the interaction between condition and angle. Comparison between the deficient conditions with and without the brace was the focus of the statistical results. A p value of <0.05 was considered significant.

## Results

Two of the ten specimens received showed evidence of PCL insufficiency based on posterior drawer examination, which was confirmed with gross inspection and were thus eliminated from the study. The remaining eight specimens underwent all test conditions and had an average age of 75 years (range: 64–89) and consisted of 7 male legs and 1 female leg.

### Force

**Fig. 2:**
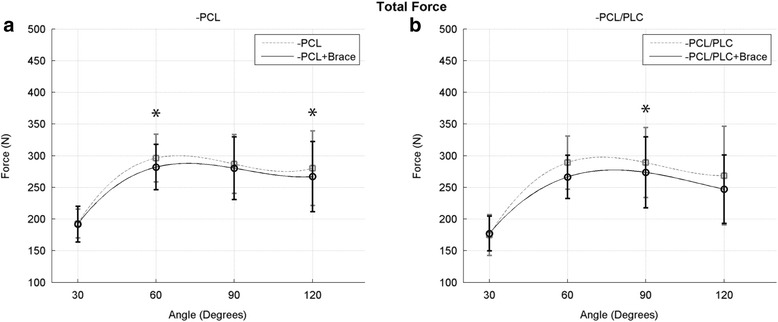
Force in the PFJ as a function of knee flexion angle in the PCL-deficient state (**a**) and PCL/PLC-deficient state (**b**) with and without the use of a dynamic brace (*indicates *p* < 0.05)

**Fig. 3:**
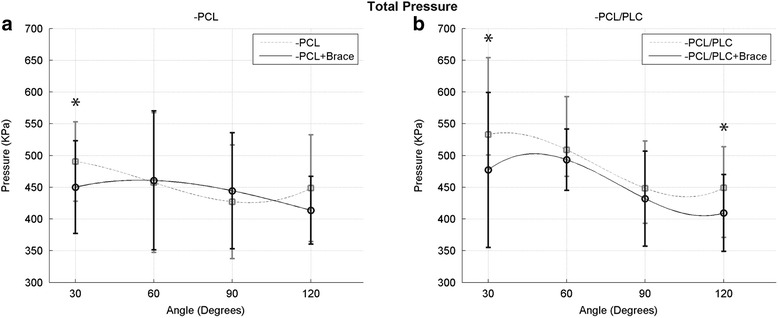
Total pressure in the PFJ as a function of knee flexion angle in the PCL-deficient state (**a**) and PCL/PLC-deficient state (**b**) with and without the use of a dynamic brace (*indicates *p* < 0.05)

Total force measured across all test conditions was lowest at 30° of knee flexion with a significant increase to 60° (*p* < 0.05), leveling off from 60° to 120° (Fig. 3). When analyzed across all angles, force through the PFJ was significantly reduced in PCL-deficient knees when a dynamic brace was applied to the extremity (*p* < 0.001) (Fig. 2a). This reduction was most significant at 120° (280.3 ± 58.9 vs 266.9 ± 55.6 N, *p* < 0.01). Likewise, across all angles tested, use of a dynamic brace in PCL/PLC-deficient knees significantly reduced PFJ force when compared to unbraced PCL/PLC-deficient knees (*p* < 0.05) (Fig. 2b).

### Total pressure

Total pressure measured within the PFJ, analyzed across all angles tested, was significantly reduced with use of a dynamic brace in both PCL- (*p* < 0.05) and PCL/PLC-deficient (*p* < 0.01) knees.

Analysis at each specific angle was also performed. PCL-deficient knees at 30° of knee flexion averaged 490.5 (±62.6) kPa, which was significantly reduced to 450.1 (±73.1) kPa with the use of the dynamic brace. At higher angles of flexion, no significant differences in total pressure between PCL-deficient knees with and without the brace were observed (Fig. 3a).

Following resection of the PLC (−PCL/-PLC), total pressure was reduced for all flexion angles tested with the addition of the dynamic brace, reaching significance at 30° and 120° (*p* < 0.05) (Fig. 3b).

### Peak pressure

**Fig. 4:**
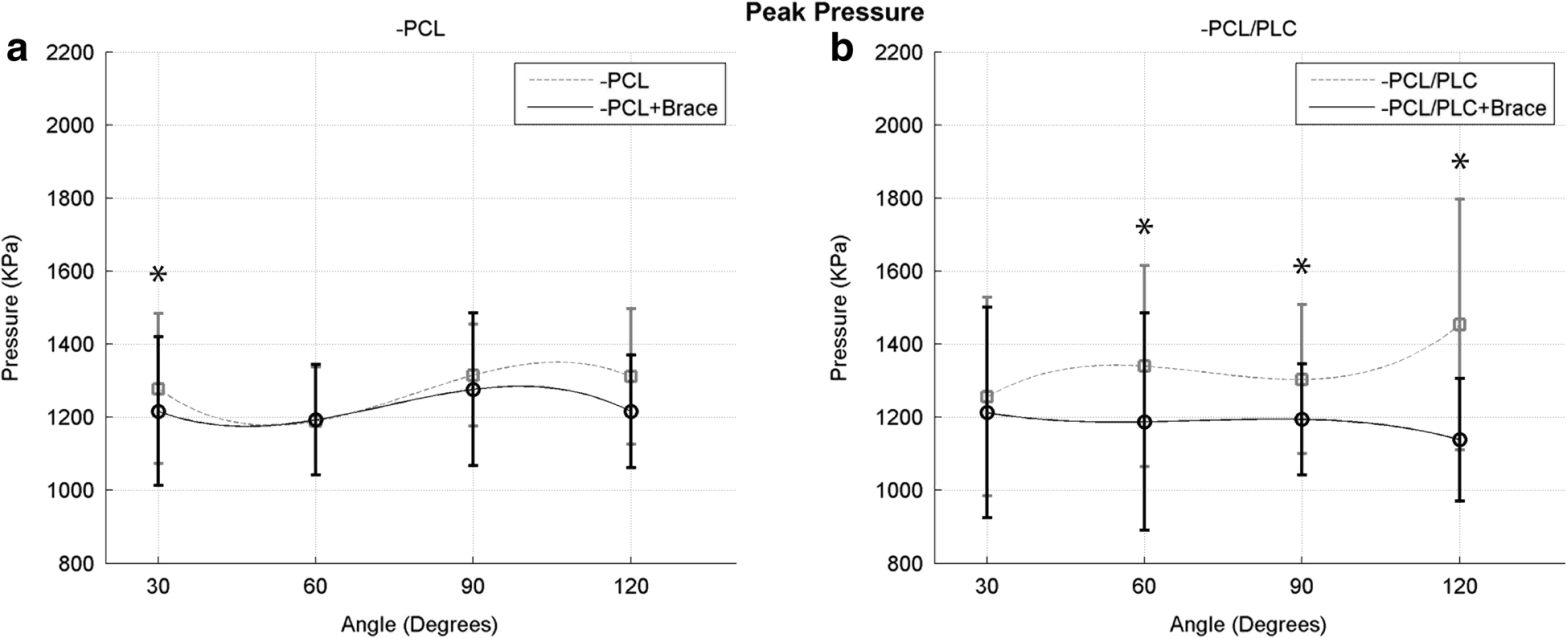
Peak contact pressure in the PFJ as a function of knee flexion angle in the PCL-deficient state (**a**) and PCL/PLC-deficient state (**b**) with and without the use of a dynamic brace (*indicates *p* < 0.05)

The overall interaction between peak contact pressure and flexion angle was not significant. When analysis was performed independent of knee flexion angle, PCL-deficient knees without a brace had a significantly higher peak pressure when compared to braced knees (*p* < 0.05) (Fig. 4a). Likewise, when analysis was performed independent of flexion angle, PCL/PLC-deficient knees without a brace had a significantly higher peak pressure when compared to braced knees. Application of a dynamic brace to PCL/PLC-deficient knees also led to a reduction in peak PFJ pressures at certain specific angles, reaching significance at 60° (1340 ± 276 vs. 1187 ± 298 kPa with brace, *p* < 0.05), 90° (1304 ± 204 vs. 1194 ± 152 kPa with brace, *p* < 0.05), and 120° (1453 ± 344 vs. 1138 ± 168 kPa with brace, *p* < 0.05) of knee flexion (Fig. 4b).

## Discussion

The most important findings in this study were that the application of a dynamic PCL brace led to a significant reduction in force, total pressure, and peak pressures in the PFJ in PCL- and PCL/PCL-deficient knees, most significantly at higher degrees of flexion. These results confirm our hypothesis that the peak pressure inside the PFJ would change more dramatically at higher degrees of knee flexion because the dynamic brace is designed to impart a larger anteriorly directed force on the tibia in that state. These results are clinically relevant because maximum posterior knee instability in PCL and PCL/PLC- deficient knees occurs immediately after toe-off with the knee in deep flexion (Iwata et al. 2007).

Previous investigators have measured contact pressures in the PFJ in PCL- and PCL/PLC-deficient knees (Skyhar et al. 1993; Gill et al. 2003a; Spiridonov et al. 2011). Both Skyhar et al. and Gill et al. reported increased PFJ contact forces in PCL- and PCL/PLC-deficient knees when compared to the intact state under simulated muscle loads at all knee flexion angles. Altered peak pressures in PCL- and combined PCL/PLC-deficient knees are most likely a result of abnormal knee kinematics. In PCL/PLC-deficient knees, the tibia translates posteriorly and externally rotates with the application of a simulated load. External rotation of the tibia leads to lateralization of the patella, which creates increased compression between the lateral facet of the patella and lateral trochlea (Gill et al. 2003a; Kwak et al. 2000). This phenomenon correlates well with our data, as peak pressures in the PCL/PLC-deficient knees were consistently isolated to the lateral facet, particularly at higher degrees of flexion.

Although previous studies have demonstrated that the use of static braces following PCL reconstruction improves posterior knee laxity (Ahn et al. 2011; Jung et al. 2008; Spiridonov et al. 2011), Jacobi demonstrated posterior laxity was not restored to the intact state (Jacobi et al. 2010). Further, Laprade demonstrated that forces applied by a dynamic brace were significantly larger than those applied by a static brace at higher flexion angles in PCL-deficient knees (LaPrade et al. 2015b). Therefore, as demonstrated by Laprade in his study, our results suggest that dynamic bracing may be a better option than static braces for management of chronic PCL injuries or to protect healing ligaments following surgical reconstruction of the PCL and/or PCL and PLC. Clinical studies are needed to determine whether the effect of dynamic bracing on peak PFJ pressures will result in improved patient outcomes and/or a lower incidence of arthrosis in patients with PCL and PCL/PLC injuries.

This study, like many cadaveric studies, is presented with several limitations. First, an axial load was not applied to the tibia. As a result, closed chain exercises that place maximum stress on the PCL, such as lunges and squats, were not properly represented. Based on the design of this brace, it was hypothesized that it could provide an even greater reduction in peak pressure during these types of exercises. A second limitation was the application of a constant hamstring and quadriceps load to each specimen in all conditions at all degrees of knee flexion. While a 2:1 *ratio* of quadriceps to hamstring loading has previously been validated (Li et al. 2003; Li et al. 2002), these forces are significantly lower than those that occur in vivo. Moreover, quadriceps and hamstring forces vary with different exercises and in different degrees of knee flexion. Nevertheless, the observed trends in peak pressure likely reflect the effect of PCL and PCL/PLC deficiency on peak pressures and how those pressures change when the knee is stabilized with a dynamic brace. Another limitation was the Tekscan sensor’s sensitivity, which, as has been previously reported (Wilharm et al. 2013), decreases with time and after multiple cycles. Shear stress, moisture, and temperature fluctuations have all been implicated as a source of sensor deterioration (Anderson et al. 2003; Jansson et al. 2013b). These effects were minimized by covering the sensor with vinyl laminate, which resists shear and water damage. In addition, the sensors were sutured in place to further minimize shear stress. A final limitation of this study is that the average age of the specimens was 75 tears old. The effect of the dynamic brace on older specimens may not accurately reflect the effect of the brace on the typical younger patient with a PCL- or PCL/PLC-deficient knee.

## Conclusions

In conclusion, the results presented in this cadaveric study demonstrate that dynamic bracing reduces force, total pressure, and peak pressure in the PFJ in PCL- and PCL/PLC-deficient knees, most significantly at higher degrees of knee flexion. While further clinical research is necessary, dynamic bracing may provide a non-invasive means to reduce the incidence of knee arthrosis in patients with PCL and combined PCL/PLC injuries.
